# Scrotal Necrosis Following Heated Intra-peritoneal Chemotherapy: Case Report and Review of the Literature

**DOI:** 10.7759/cureus.20638

**Published:** 2021-12-23

**Authors:** Samiha N Fagih, Rana M Baghdadi, Aeshah Y Banjer, Amal A Ismail, Majda A Addas, Alaa A Shabkah, Nora H Trabulsi

**Affiliations:** 1 Department of Surgery, King Abdulaziz University Faculty of Medicine, Jeddah, SAU; 2 Department of Surgery, International Medical Center, Jeddah, SAU

**Keywords:** hyperthermic intraperitoneal chemotherapy, colorectal cancer, cytoreductive surgery (crs), cytoreductive surgery and hipec, scrotal edema

## Abstract

Scrotal necrosis is a rare occurrence that is scarcely reported among patients having undergone heated intra-peritoneal chemotherapy (HIPEC) procedures. Due to anatomic factors and the thermally enhanced cytotoxicity of chemotherapeutic agents, this complication can have debilitating post-operative effects. We herein highlight the presentation of scrotal necrosis in a patient who underwent HIPEC procedure for peritoneal metastasis secondary to colorectal carcinoma, and how it contrasts to previously documented cases of a similar nature. Furthermore, we describe a successful management strategy that consisted of conservative measures followed by surgical debridement and primary repair, and enabled the patient to experience significant functional and cosmetic improvement.

## Introduction

Heated intra-peritoneal chemotherapy (HIPEC) is utilized in the management of disseminated intra-abdominal malignancies, such as colorectal [1], ovarian [2], appendiceal [3], and primary peritoneal neoplasms. It entails regional infusion of heated chemotherapy in adjunction to cytoreductive surgery (CRS). The chemotherapeutic agent acts synergistically with heat to target microscopic disease after the macroscopic tumor has been surgically excised. This has been shown to improve long-term disease-free and overall survival [4]. Mitomycin C (MMC) is frequently used in this context [5,6]. Despite its favorable profile, MMC is linked to side effects such as neutropenia (40%) [7], respiratory complications (17%), intra-abdominal collections (8.8%), anastomotic leaks (4.4%), wound infections (7.2%), ileus (6.2%), and acute renal injury (5.6%) [8]. A rare yet important complication is scrotal pain and ulceration after MMC administration in HIPEC. Only a few recorded cases have been described in the literature [9-13]. This is a case report of scrotal necrosis after HIPEC for adenocarcinoma of the colon with peritoneal metastasis (PM).

## Case presentation

A 39-year-old man presented with PM secondary to colorectal carcinoma. He had initially undergone an extended right hemicolectomy followed by adjuvant oxaliplatin and capecitabine (XELOX). Subsequent surveillance imaging revealed suspicious peritoneal deposits confirmed to be malignant through diagnostic laparoscopy and peritoneal biopsy. He underwent CRS including low anterior resection for a large infiltrative pelvic deposit, seminal vesicles resection, and peritonectomy along with MMC-based HIPEC. 

A small number of pelvic ascites was noted intra-operatively. Cytological analysis of the fluid was obtained and showed malignant cells. This finding alongside the patient’s clinical background and peritoneal cancer index (PCI) score of 11, led to the decision to proceed with HIPEC. MMC was administered at 41^o^C for 90 minutes. The surgery was well-tolerated by the patient. His post-operative course was complicated by a urinary tract infection and paralytic ileus, which resolved shortly thereafter. 

**Figure 1:**
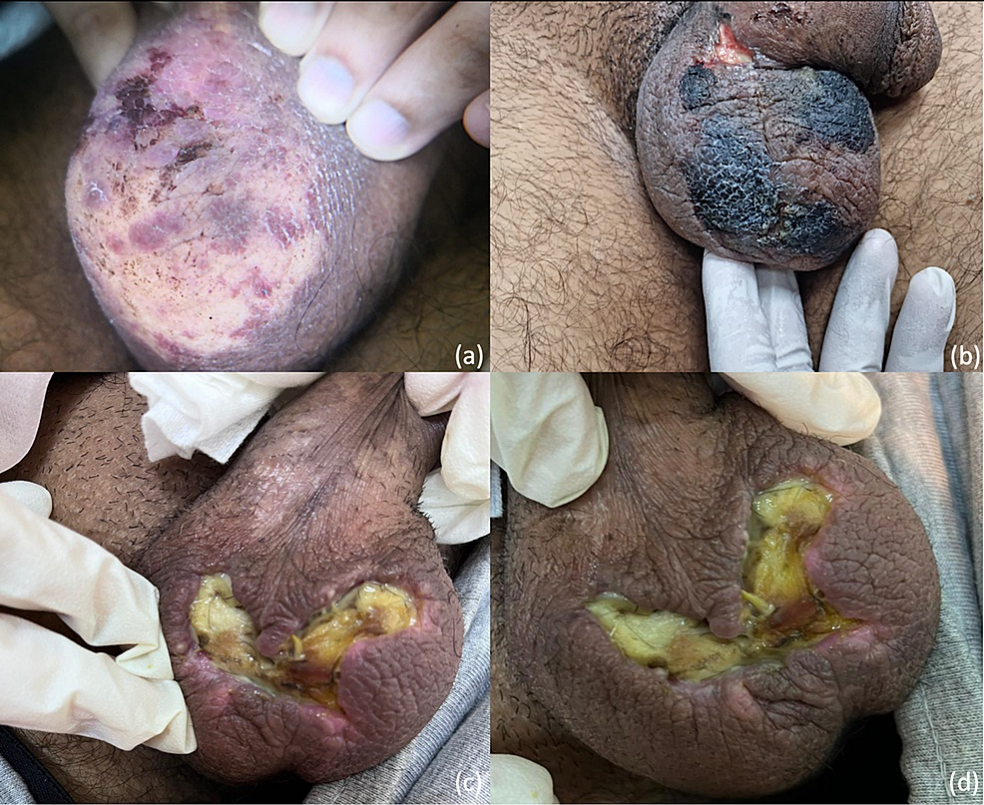
Progression of scrotal necrosis post mitomycin C based heated intra-peritoneal chemotherapy (HIPEC) The image is showing (a) early changes on anterior scrotal skin, (b) progression of scrotal necrosis, (c) exposed subcutaneous necrotic tissue, and (d) final stage prior to debridement and primary closure.

On post-operative day 30, he began to develop scrotal swelling and discoloration in the form of diffuse erythema with black spots (Figure 1, panel a). He was seen by the urology team, who recommended watchful waiting. His symptoms continued to progress. On post-operative day 52, he presented to the emergency department with worsening ulceration. He reported burning scrotal pain that radiated to his medial thigh and affected his mobility. Examination revealed scrotal tenderness with mild swelling. The overlying skin was erythematous with signs of eschar formation. Three necrotic demarcations were noted on the scrotum with ulceration associated with purulent discharge at the scrotopenile junction (Figure 1, panel b).

**Figure 2:**
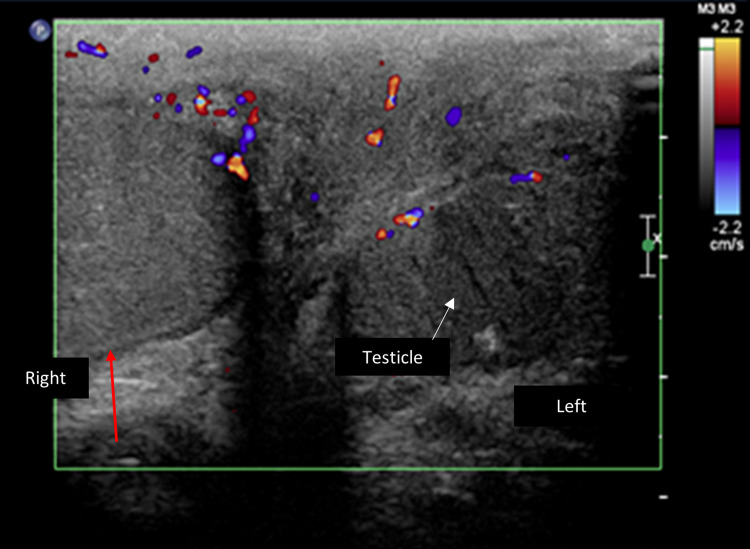
Scrotal ultrasound ruled out underlying collections. The image is showing subcutaneous thickening and increased subcutaneous vascularity suggestive of scrotal cellulitis (red arrow: subcutaneous thickening; white arrow: left testicle).

**Figure 3:**
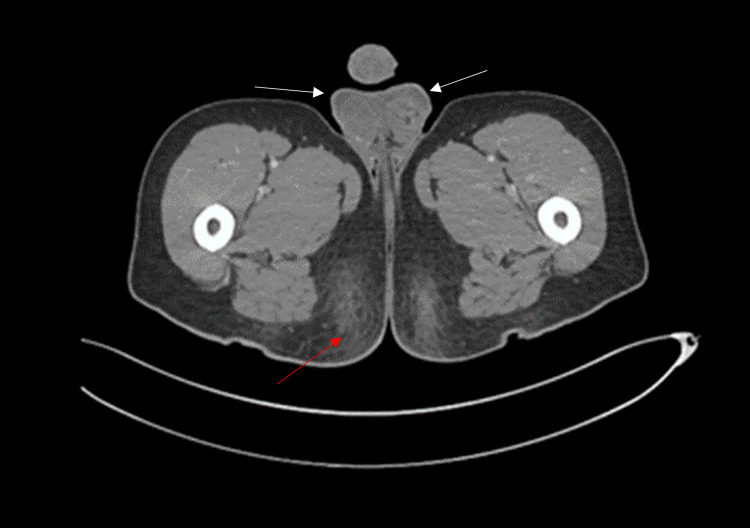
Axial view of computed tomography of the pelvis with IV contrast demonstrating diffuse subcutaneous edema with scrotal wall thickening bilaterally. No soft tissue/fat stranding, fascial thickening nor free gas was noted (red arrow: diffuse subcutaneous edema; white arrow: scrotal wall thickening bilaterally).

Upon admission, the patient’s biochemical profile was unremarkable with normal cell counts and coagulation panel. Scrotal ultrasound ruled out underlying collections (Figure 2). Kidney-ureter-bladder ultrasound was unremarkable. Computed tomography of the pelvis ruled out recurrence and necrotizing fasciitis as well as deep collections (Figure 3). 

The urology service was consulted. Their impression was that of gangrenous cellulitis, for which they recommended conservative management with antibiotics and local wound care. The patient received systemic and topical antibiotics with broad-spectrum and anti-fungal coverage (meropenem, vancomycin, fluconazole, and topical silver sulfadiazine). Antibiotics were introduced on an empirical basis based on the clinical picture of purulent discharge. However, following a negative tissue culture result, we discontinued the antibiotics. Moreover, blood and urine cultures were negative for any growths and serological studies failed to identify an infectious process. The symptoms persisted and the scrotal lesions progressed (Figure 1, panel c). 

Further consultations were sought from the dermatology service. Their differentials included gangrenous cellulitis, chemotherapy-induced vasculopathy, and pyoderma gangrenosum (PG), for which he was started on a regimen of high-dose topical steroids cream and flamazine. The patient subsequently began to demonstrate improvement within the following weeks. Figure 1, panel d, documents the resolution of the eschar with visible, well-demarcated necrotic tissue. 

**Figure 4:**
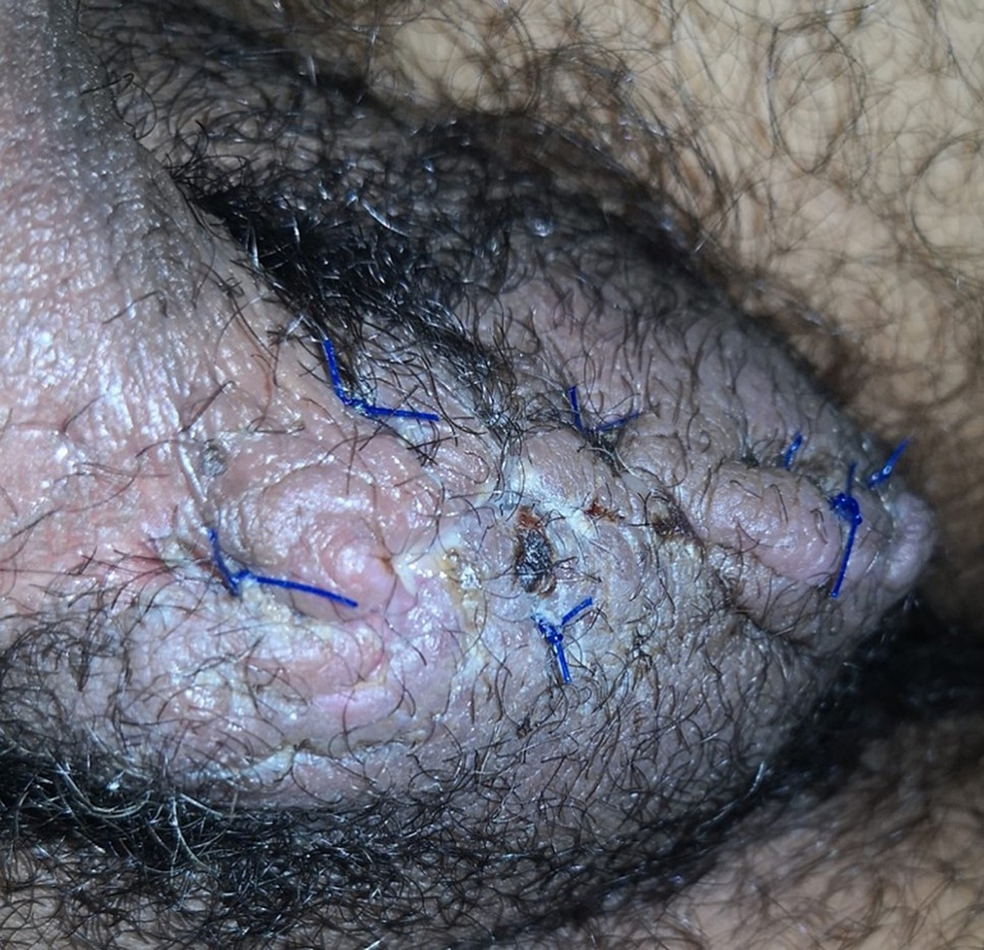
Post-surgical debridement and primary repair.

Meanwhile, the patient required multiple lines of narcotics for concomitant pudendal neuropathy. He eventually required an impar ganglion block by pain service. An anesthetic substance (bupivacaine and 100% alcohol) is injected into the impar ganglion, which is positioned anterior to the sacrococcygeal joint. This resulted in a progressive reduction in his perineal pain and narcotic usage. On post-operative day 95, the patient underwent surgical debridement and primary repair (Figure 4). Bilateral viable testicles were observed intra-operatively. Histopathological analysis showed mixed inflammatory and reactive changes.

## Discussion

Necrosis of the genitals is an infrequent yet devastating consequence of HIPEC. It manifests as the pain of the genitalia with apparent tissue devitalization during the post-operative phase. This is postulated to occur due to the underlying anatomy of patent processus vaginalis (PPV), which allows the chemotherapeutic agent to travel into the intra-scrotal region from the peritoneal cavity [9,10]. When this occurs intra-operatively, the scrotum cannot be evacuated simultaneously with the peritoneal cavity, leading to extended contact with MMC. As a result, the principle of hyperthermia-enhanced tissue penetration and cytotoxicity of MMC which makes HIPEC an effective treatment solution raises the risk of instigating a massive inflammatory response with resultant ulceration and necrosis. 

The literature documenting this complication is limited. Table 1 summarizes the clinical data from five case reports [9-12], and one case series published by Baron et al. based on a total of 13 cases retrospectively identified from two specialized centers [13]. MMC was used as the intra-peritoneal chemotherapy agent of choice for all 18 cases. The clinical data portrayed by the authors are largely consistent. The patients presented similarly with pain, scrotal swelling, and skin changes that ranged from diffuse erythema to well-demarcated gangrenous eschars. Systemic response in the form of fever, sepsis, and leukocytosis was not commonly encountered or reported. The timeline of this presentation was variable among the reported cases, from days to months post-operatively. Notably, resolution of symptoms necessitated surgical management among 14 out of 18 of the cases described. 

**Table 1:** Review of literature on HIPEC-associated scrotal necrosis HIPEC: hyperthermic intra-peritoneal chemotherapy; IQR: inter-quartile range; US: ultrasound; CT: computed tomography; WBC: white blood cell

Case report									
Author/year	Age	Type of malignancy	Time of presentation	MMC infusion	Response to antibiotics	Laboratory and cultures	Imaging	Resolution	Pathology
Case 1: Akhavan et al., 2007 [9]	48	Metastatic appendiceal adenocarcinoma	4 months post-operatively	30 mg over 60 minutes at 41°C followed by 10 mg over 40 minutes	Minimal: pain mildly improved	Blood and urine cultures negative for growths	Right-sided hydrocele and testicular hyperemia with scrotal wall thickening	Surgical excision of the ulcer and surrounding necrotic tissue followed by primary repair	Necrosis and granulation
							Negative for abscess/Fournier’s gangrene		
Case 2: Akhavan, et al., 2007 [9]	66	Recurrent peritoneal mesothelioma	3 months after the second HIPEC in 2 years	30 mg over 60 minutes followed by 10mg over 40 minutes	Initial response followed by recurrence 2 months later	Cultures negative for growths	Negative for collections	Excision followed by primary repair	Inflammatory changes and reactive fibrosis
Case 3: Aziz et al., 2015 [11]	33	Pseudomyxoma peritonei of appendiceal origin	2 months (Post-operative Day 67)	18 mg over 60 minutes at 42°C	Not mentioned	Cultures negative for growths	Scrotal skin thickening	Excision followed by primary repair	Not mentioned
							Negative for collections		
Case 4: Fabiana et al., 2012 [10]	65	Metastatic rectal adenocarcinoma	9 days	Not specified	No response	Cultures negative for growths	Negative for collections	Wound debridement and primary repair	Ischemic necrosis on punch biopsy
Case 5: Bartlett et al., 2019 [12]	54	Metastatic appendiceal carcinoid adenocarcinoma	3 months	Not specified	Not mentioned	Not mentioned	CT pelvis showed bilateral hydroceles without abscess or collection	Initial improvement with topical 60% dimethyl sulfoxide (DMSO) followed by debridement and partial scrotal resection	Epidermal necrosis without evidence of vasculitis
							US showed scrotal thickening without masses or collections		
Case series									
13 patients reported by Baron et al., 2021 [13], from two peritoneal malignancy centers with a total of 1597 HIPEC	Median age: 57 (﻿IQR: 49-64)	8 (62%) appendiceal, 3 (23%) Colon, 1 (8%) gastric tumors, and 1(8%) mesothelial cysts. (histological subtypes not specified)	Median: 64 days ﻿(IQR: 60-108)	40 mg in 11/13 patients, 28 mg in 1/13 patient and 22 mg in 1/13 patient	Trial of conservative therapy with antibiotics in 8/13 of patients with inadequate response	Median cell counts at time of genital necrosis	Hydrocele (n=3), labia tissue edema (n =1), scrotal wall thickening (n=3), and subcutaneous emphysema (n =1)	Surgical debridement was performed in 9/13 (70%) cases	Pathology reports were available for 5/9 of the patients that underwent debridement
						WBC (×10^9^/L): 9.0 (7.2-12.5)			
				Perfusion was over 90 minutes in 11/13 patients and over 60 minutes in 2/13 patients		Hemoglobin (g/dL): 11.3 (9.8-12.8)	Absence of testicular involvement, abscess, or compromised blood flow in all cases	Median time between presentation and surgical treatment of 57 days (IQR: 8-180)	Skin and subcutaneous necrosis
						Platelets (×10^9^/L): 457 (369-551)			

This case is unique in its relatively early presentation following HIPEC. Whether this is due to patient-related or procedural factors has not been determined. Moreover, the rate of progression of the lesion and its positive response to steroids have not been described elsewhere. Other authors have documented minimal to partial improvement of symptoms with systemic antibiotics [9,13]. Fabiana et al. also reported partial improvement with topical scavenger agent dimethyl sulfoxide [10]. While this approach was deemed inadequate, Baron et al. highlighted the significance of early conservative measures to prevent premature operative management [13]. The anti-inflammatory effects of the aforementioned agents may mitigate the response elicited by MMC and allow the lesions to demarcate prior to surgical excision. This is supported by two cases that were prematurely excised and complicated by impaired wound healing and secondary necrotic lesions [13]. 

Some authors have suggested screening for PPV with pre-operative imaging or intra-operative identification to prevent this complication [10,11]. The underlying anatomy of PPV has been extensively studied in the context of peritoneal dialysis patients presenting with scrotal edema and post-prostatectomy patients presenting with inguinal hernia [14,15]. The radiological demonstration of PPV via radionucleotide scintigraphy and CT peritoneography has been used to predict genital edema among peritoneal dialysis patients [14,15]. Lee et al. described intra-operative identification of PPV by looking for a dimple with or without an associated visible canal towards the deep inguinal ring [16]. Others have extrapolated his findings by exploring methods of PPV occlusion to prevent inguinal hernia post-prostatectomy [17,18]. To our knowledge, none of these approaches have been performed in HIPEC procedures. 

## Conclusions

More cases should be recorded to ensure a better understanding of the etiology and natural course of this complication and to determine which populations are at a higher risk of developing it. Moreover, it is vital to promote awareness among surgeons who may incorporate this into their pre-operative counseling for patients undergoing HIPEC procedures.
